# RNA Viruses and RNAi: Quasispecies Implications for Viral Escape

**DOI:** 10.3390/v7062768

**Published:** 2015-06-19

**Authors:** John B. Presloid, Isabel S. Novella

**Affiliations:** Department of Medical Microbiology and Immunology, College of Medicine, The University of Toledo, 3055 Arlington Avenue, Toledo, OH 43614, USA; E-Mail: John.Presloid@utoledo.edu

**Keywords:** RNA virus, RNAi, escape, adaptations, quasispecies

## Abstract

Due to high mutation rates, populations of RNA viruses exist as a collection of closely related mutants known as a quasispecies. A consequence of error-prone replication is the potential for rapid adaptation of RNA viruses when a selective pressure is applied, including host immune systems and antiviral drugs. RNA interference (RNAi) acts to inhibit protein synthesis by targeting specific mRNAs for degradation and this process has been developed to target RNA viruses, exhibiting their potential as a therapeutic against infections. However, viruses containing mutations conferring resistance to RNAi were isolated in nearly all cases, underlining the problems of rapid viral evolution. Thus, while promising, the use of RNAi in treating or preventing viral diseases remains fraught with the typical complications that result from high specificity of the target, as seen in other antiviral regimens.

## 1. RNA Virus Replication and Evolution

**Figure 1:**
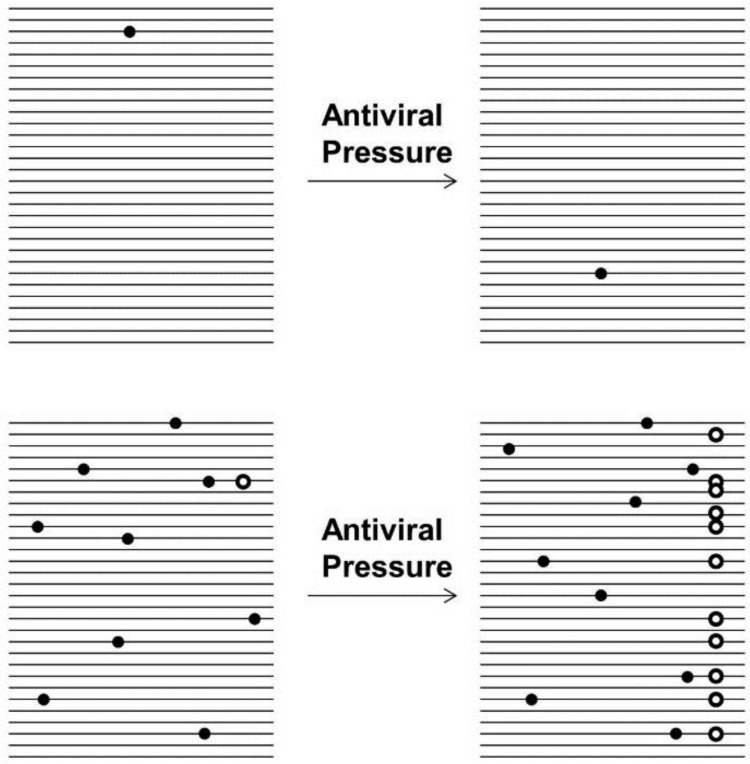
**Virus quasispecies**. A representation of mutation differences between DNA species (top panel) and an RNA virus quasispecies (bottom panel). Each line represents a portion of the genome, and each circle is a randomly-generated mutation. Each mutation will have a random effect, with most mutations being deleterious or neutral (dark circles), but with a small chance to disrupt targeted interactions by drugs, antibodies, or RNAi (white cirlces). In the DNA population on top, there is very little genetic variation, and thus it would be expected to have a nearly uniform phenotype in most environments. The RNA quasispecies on bottom however contains many unique random mutations, each of which will have an unknown effect. If the mutations happen to be in a gene targeted by a specific antibody or drug, then that virus could be resistant, and would be favored to multiply and increase its proportion in the quasispecies, creating a larger pool of resistant viruses.

One major factor in the evolutionary success of RNA viruses is the utilization of error-prone replication that generally lacks proofreading mechanisms [1,2]. Average mutation rates are about 10^−4^–10^−5^ errors per nucleotide copied or, based on average genomic sizes, about one mutation per genome copied. As a consequence a virus population exists not as an assembly of genetically-identical sequences, but rather a collection of closely-related mutants termed a quasispecies [3,4,5,6], represented in Figure 1. Quasispecies theory holds important repercussions for RNA virus evolution and adaptability. As a large population of randomly generated mutants, a quasispecies viral population will have a wide range of phenotypic variation. Genetic recombination provides additional sources of genetic variation. In positive stranded viruses copy-choice provides a mechanism of homologous recombination [7]. Viruses with segmented genomes, such as influenza virus, can undergo reassortment. During reassortment, coinfection of multiple viruses results in genomic segments from different infecting viruses mix in a single cell, which are then free to be assembled in any combination in the resulting progeny viruses. Having a large amount of pre-existing variation would then lead to rapid adaptation in response to changes in the environment [8,9], and the viral quasispecies can already contain resistant mutations even before a selective pressure is applied [10]. The high mutation rates found in an RNA quasispecies also increase the probability of the generation of escape mutations. These escape mutations would then be selected for in the presence of environmental pressures (*i.e*., drugs or antibodies). This means that a quasispecies is both more likely to already contain random resistance mutations, and also more likely to generate them during replication. Thus it is important to consider the quasispecies nature, and thus the presence of a low level of random mutations, when applying any sort of antiviral therapeutic.

An important concept in viral evolution is the Red Queen Hypothesis [11]. In Lewis Carroll’s “Through the Looking-Glass”, the Red Queen states “It takes all the running you can do, to keep in the same place.” In evolutionary biology, Red Queen may apply to competing populations or to prey-predator (or virus-host) interactions. In the case of competing populations coexistence is not the result of evolutionary stasis. Instead populations are constantly adapting and improving to the environment, but because competitors evolve at the same speed it is a zero-gain game. In the case of virus-host interactions coexistence it is the result of an arms race between the two, such that for every new phenotype that one partner develops to attack the other partner responds with a counterattack. Indeed, a viral quasispecies of sufficient size would be likely to be able to produce random escape mutations very rapidly [12]. A well-known example of clinical importance is the capacity of an RNA virus to escape immune targeting, followed by a new immune response that can be, in turn, followed by a new round of selection for escape mutants [13,14]. This is a common hallmark of RNA viruses and has been extensively documented in clinically relevant viruses as diverse as influenza [15,16,17], hepatitis C [18,19], and HIV [20,21,22]. Immune evasion is also important with regards to vaccines, which depend on antibody production and immune activation in order to protect against viral infections. Indeed, in accordance with the Red Queen Hypothesis, the struggle to develop consistently effective vaccines for many RNA viruses continues to be a difficult challenge [23,24,25,26].

## 2. Escape from Antiviral Drugs

With a better understanding of how viruses replicate, a variety of different antiviral drugs have been developed to help combat infections. The development of resistant viruses also has been observed for many antiviral drugs and their respective viral targets. There are two types of drugs targeting influenza: M2 inhibitors and neuraminidase inhibitors. Amantidine and rimantidine target the M2 ion channel, but overuse in farming has resulted in the selection of resistant mutants and these drugs are no longer effective [27]. There are several neuraminidase inhibitors, but resistance can develop easily. For example, multiple mutations have been identified in different influenza strains worldwide that cause these viruses to be resistant to oseltamivir (Tamiflu™), the most commonly used anti-influenza drug [28,29,30]. Although many of the observed mutations lower replicative fitness in these resistant viruses, secondary compensatory mutations can eliminate fitness losses while also retaining drug resistance [31]. Oseltamivir-resistant strains of influenza have recently been identified in reservoir bird populations as well, even in the absence of continuous selective pressure, which could prove to be troubling if it leads to newly-emerging resistant strains [32]. Additionally, mutations which confer drug resistance have been studied and mapped extensively in HIV against a large array of antiviral drugs [33], including resistance to the relatively new small interfering peptides [34]. Administration of multiple drugs simultaneously has helped reduce and delay the development of resistance, but is not enough to prevent this completely and resistant mutations still arise in the population [35]. However, the cumulative fitness cost of resistance to each of the drugs in the cocktail ensures that virus replication is minimized to levels that are frequently undetected, making combinational therapy very successful.

Some antivirals act broadly. One example is ribavirin, a purine analog that has multiple targets in the replication cycle [36]. Ribavirin has equal affinity for cytosine and uracil, which causes mismatches when replicating these nucleosides and thus increases the mutation rate of replicating viruses, leading to a loss of functional progeny [37]. This lack of specificity might suggest that resistance to ribavirin would be difficult to achieve, but ribavirin-resistant mutants were soon discovered in poliovirus experiments [38,39], highlighting the fact that viruses are quickly able to adapt to even severely mutagenic drugs. Ribavirin resistance can be due to a single nucleotide substitution that results in a polymerase with increased fidelity, and it is well-documented in many other different RNA viruses, including foot-and-mouth disease virus (FMDV) influenza and hepatitis C (reviewed in [40]). Ribavirin resistance in hepatitis E virus (HEV) *in vivo* has been linked to a single nucleotide mutation in the polymerase region of the genome, although the exact mechanism of this mutation is still unknown [41]. Additionally, ribavirin was also found to deplete intracellular GTP levels *in vitro*, giving it an additional mechanism of action to restrict replication of HEV [42].

## 3. A New Approach to Antiviral Intervention

The lack of antivirals against many viral diseases and the development of resistance to current treatments make the search for alternatives options a priority. One newer such alternative of great interest is the development of RNA interference (RNAi) systems. First observed in *C. elegans* [43], RNAi refers to a process by which cells or viruses produce small single-stranded segments of RNA called microRNA (miRNA) to serve as guiding templates for recognition of specific mRNAs (reviewed in [44]). While this can have several different effects, the most commonly understood effect is the subsequent destruction of the targeted mRNA, and thus an inhibition of protein synthesis, which is referred to as “gene silencing”. RNAi was shown to inhibit an RNA virus soon after its initial discovery [45], opening a door to a novel form of RNA virus treatment. An ideal RNAi treatment of viruses holds a possible advantage over the use of antiviral drugs in that the regimen would be expected to be much simpler and easier to follow. Rather than having to adhere to a strict schedule of taking a number of drugs on a continual basis, the ideal RNAi treatment plan could involve only a small number of treatments to provide long-lasting inhibitory effects.

Since this discovery the literature has flourished with studies testing the antiviral efficacy of RNAi *in vitro*. Synthetic miRNA that targets specific viral sequences, termed siRNA, can be created in the laboratory, preferably selecting conserved regions [46]. This technique was used with the New Guinea C strain of Dengue virus 2 (DENV2) to produce several different siRNA products. The uptake of siRNA into BHK host cells caused a variable increase in cell survival when challenged with a DENV2 infection, and a decrease in viral titer recovered from media [47]. This inhibition improved when the siRNA was delivered via lentivirus instead of transient uptake, which suggests that the type of delivery method is an important factor in the design of any potential RNAi therapeutic. Later studies demonstrated the efficacy of RNAi in inhibiting a wide variety of viral infections, including hepatitis C [48,49,50], FMDV [51,52], tick-borne encephalitis virus [53], influenza virus [54,55], and HIV [56,57,58]. In all of these studies, RNAi was designed to target viral mRNAs, and then applied, either through transient uptake or by using a lentivirus, to a variety of cells cultured *in vitro*. The RNAi treatment provided a range of virus suppression, showcasing the potential of RNAi to be effective in treating a broad array of different RNA viruses.

Another method of RNAi treatment is to target cellular accessory or entry proteins that are used by the virus during infection. By targeting host cells, researchers hope to use more mutationally stable targets instead of the rapidly mutating viral proteins. For example, researchers have been able to design RNAi against human ST6GAL1, which helps form sialic acid receptors on cells which serve as an important influenza receptor. This approach resulted in increased resistance to influenza attachment and thus infection [59]. Another study found that siRNA against CCR5, an HIV cellular coreceptor, led to a suppression of HIV replication in RNAi-treated macrophage cells [60]. Similarly, researchers identified an array of HIV cellular cofactors, and then engineered siRNA to specifically target their respective cellular mRNAs. HIV replication was significantly inhibited in half of the treated cell lines that were tested, which further supports the promise of this method [61]. However, and not unexpectedly, several of these siRNA treatments also exhibited toxic effects on the cells, underlining the importance of careful design while being sensitive to potential adverse effects.

The initial successes of *in vitro* studies led to the performance of extensive work *in vivo* that confirmed the efficacy of RNAi used against viruses. RNAi is effective in protecting mice from influenza infection both when administered through retroorbital injection [54] or intranasally [62]. Interestingly, experiments performed with Ebola virus found that application of siRNA postexposure can be effective in preventing lethality both in guinea pigs [63] and macaques [64]. This is important because it represents a postexposure model to prevent lethality, which would be useful in the field after known exposures to help prevent further progression of disease and symptoms. Additional work has been done testing the efficacy of RNAi treatment on humans. Clinical trials using an intranasally administered dose of siRNA targeting the nucleocapsid was able to decrease the incidence of respiratory syncytial virus infection upon inoculation in a double-blind study on humans [65]. A similar study carried out using an orally administered RNAi therapeutic also found a decrease in viral load among individuals treated across a wide range of doses; however they also found a handful of incidents of minor adverse reactions [66]. Other clinical trials are reviewed in [67,68,69,70], although it must be noted that many trials were terminated due to a lack of efficacy or a prevalence of adverse effects such as increased inflammation. One of the major issues of translating RNAi research into *in vivo *models has been the difficulty associated with delivering the RNAi into the appropriate tissues and cells in the body. For example, RNAi injected directly into the brain of mice is effective in suppressing both West Nile virus (WNV) and Japanese encephalitis virus (JEV), two flaviviruses that can target neurologic tissues [71]. While these results were encouraging, in order to be clinically relevant a more convenient delivery method would be necessary, as cranial injections are not realistic and the blood-brain barrier prevents standard intravenous injections from reaching neural tissues. One model solved this issue by pseudotyping a lentivirus vector with rabies glycoprotein, which displays neurospecificity and is able to naturally cross the blood-brain barrier. Doing this allowed neural targeting and protection in mice even when injected distantly in the tail vein [72]. While treatment with antiviral drugs is capable of decreasing viral loads by many logs during an active infection (reviewed in [73]), there are few long-term studies analyzing the viral loads seen in RNAi treaments *in vivo*. RNAi treatment *in vivo* has shown decreases in viral loads of 2–3 logs of hepatitis B virus (HBV) in mice after 21 days. Although titers gradually recovered to within a log of untreated mice over 120 days, they were still lower than untreated mice [74]. A similar gradual recovery of titer is seen when using RNAi to protect swine against porcine reproductive and respiratory syndrome virus (PRRSV) [75], suggesting that other measures are necessary to confer long-term reductions of viral loads during infection.

## 4. Beyond siRNA: The Problem with Resistance

With the apparent potential of RNAi for the treatment of viral diseases, it is of vital importance to be aware of potential issues and difficulties. In addition to the typical issues of human therapeutics, namely safety and efficacy, any targeting of RNA viruses will necessarily require a great deal of effort to combat the natural capacity of the viruses to evade inhibitory actions. Quasispecies theory predicts that a replicating viral population will contain a large number of unique mutants, so there is a probability that an active viral infection will already have resistant mutants upon time of RNAi treatment, which could rapidly become the dominant mutation when placed under selection. Similarly, the mutation could occur once under the selective pressure of the RNAi, especially if the initial pressure is not strong enough to fully inhibit all viral replication. Thus, there is little reason to believe that viruses would be less able to mutate to evade RNAi than they have proven against other antagonists. Indeed, there are already multiple confirmations that RNA viruses are capable of producing RNAi escape mutants. Often, these mutations are in the sequence which is directly targeted by the siRNA, as seen with poliovirus [76], JEV [77], hepatitis C virus [78], turnip mosaic virus [79] and the model morbillivirus peste des petits ruminants virus (PPRV) [80]. The mechanism for this type of escape mutant is easiest to explain, as RNAi requires stringent sequence agreement in order to operate effectively. Thus given the quasispecies nature of the virus population and its propensity to adapt rapidly in the presence of pressure, it would be expected to develop and propagate mutants which were no longer able to be recognized by siRNA. Similar results are also extensively documented in RNAi targeted against HIV. Numerous studies have found that HIV escapes siRNA designed to target conserved regions such as the *tat *gene [81], the *nef* gene [82], and the *int* and *att* genes [83].

In one particularly thorough study, multiple HIV strains were engineered to include a single mutation in the *nef* region targeted by an siRNA strand, so that there was one mutant for every nucleoside contained in the siRNA. Sixteen out of the 19 positions yielded viable virus, and 10 out of those 16 viable mutants replicated with equal efficiency both in the presence of siRNA and its absence, demonstrating that the inhibitory effects of RNAi can be completely overcome not only by a single mutation, but by a large variety of single mutations [84]. This highlights both the extreme sensitivity RNAi has for sequence identity, and also the issues faced in viral escape of RNAi. Furthermore, this study highlights the possibility that not all resistance mutations will involve fitness tradeoffs. We propose that siRNA for which tradeoffs are observed during escape should be prioritized and may be more useful, particularly if used in a cocktail.

Interestingly, resistance mutations are not always located in the target sequence, suggesting multiple mechanisms can be used to escape RNAi. In a study with FMDV, researchers were unable to find any mutations in the siRNA-targeted regions of resistant viruses [85]. While the remainder of the genomes outside the targeted region was not sequenced in this study, a reasonable inference is that these genomes developed some sort of escape mutation or mutations outside the region of interest, which decreased the effectiveness of the siRNA treatment. In another study using HIV, resistant viruses developed mutations which allowed them to escape siRNA by increasing the overall rate of viral transcription. This led to an increased amount of viral RNA, which overwhelmed the capabilities of the siRNA and making the viruses resistant to siRNA in general, even siRNA that was not used initially [86], although others argue this result is due to standard direct resistance mechanisms [87]. In HIV, another escape mechanism was observed, with mutations providing resistance to RNAi treatments by altering RNA secondary structure, rendering the siRNA sterically unable to bind the target [88]. As RNA secondary structures are sensitive, mutations which alter this structure could appear in many different places in the genome, and thus would also be more difficult to predict. A summary of studies identifying siRNA resistance mutations is provided in Table 1. Further reading on the topic of viral escape from RNAi is provided by several excellent reviews [68,89,90].

**Table 1:** A selection of studies which document mutations in RNA viruses conferring resistance to siRNA.

Virus	siRNA target(s)	References
HIV	*nef*	[75,77,85]
	*tat*	[74]
	*int*	[76]
	*att*	[76]
	Multiple siRNA, mutations outside targeted sequence	[79,81]
Poliovirus	Multiple siRNAs	[69]
Hepatitis C	Multiple siRNA	[71]
JEV	Multiple siRNAs	[70,76]
FMDV	siRNA-resistant, no mutations in targeted sequence	[78]
Turnip Mosaic Virus	HC-Pro	[72]
PPRV	Nucleoprotein	[73]

## 5. Combinational Therapy with siRNA

There has been promising research into the use of a combination of antiviral approaches, often referred to as combinatorial therapy. The addition of more stresses should make it more difficult for the viruses to be able to evade quickly, as they would be less probably to already hold the multiple mutations that would be likely necessary to successfully escape each siRNA. Experiments designed to use multiple different siRNA molecules targeting HIV [91,92] and JEV [83] found that this method is effective in providing more protection against viral infection than single siRNAs. It should be noted however that escape mutants were still found in these studies, but their presence was delayed compared to control infections. The siRNAs tested from another study exhibited antiviral activity against mutant HIV strains, but eventually selected for a new set of mutants that were able to replicate in the treated cells [93], providing another example of Red Queen dynamics. These results are important, as they imply that even multiple siRNAs might typically not be enough to completely stop replication of a virus, a theme seen earlier in testing antiviral drugs. A more promising route seems to be a multi-faceted approach in which multiple antiviral measures are administered in conjunction [94]. The use of siRNA with other antiviral drugs has proven to be effective against HIV [95]. A combination of siRNAs targeting HIV, siRNAs targeting cellular cofactors required for virus replication, and antiviral drugs exhibited a synergistic effect, providing long-lasting protection of cells against HIV [96]. However, this study did still see HIV replication occur eventually, although mutational analysis was not performed. Overall, combinational therapy using siRNA shows promise but, not unexpectedly, resistance will still be a problem. However, we have reasons to be optimistic if we can find cocktails containing drugs for which escape has such high cost that viral replication is virtually halted. One must also be aware that, as with other therapeutic methods, there remain issues of RNAi bioavailability, delivery, and specificity which are reviewed in-depth in [90,97,98]. While these issues are unrelated to quasispecies and mutational escape, they need to be under careful consideration for any successful viral treatment regime.

## 6. Conclusions

The studies highlighted in this review demonstrate the promise of RNAi in combating viral infections and diseases. The ability to specifically target viral genomes and genes has the potential to be very effective in attacking viruses. However, this specificity also results in minimal mutational requirements for escape, and the quasispecies nature of viral populations means that escape mutants will be selected. The infecting population may have a reservoir of minority mutations that can provide resistant to new therapies. Alternatively, particularly during early stages when the viral population may be very small, high mutation rates will result in a rapid *de novo* generation of new mutants. This ability to rapidly escape antiviral challenges has been well-documented both experimentally and clinically with regards to antiviral drugs, and is now beginning to be seen and understood with regards to RNAi treatments as well. While combinatorial treatments appear to hold some promise, it is important to note that escape mutants have been observed in many combinatorial drug experiments in the past. So while the potential for siRNA therapy is large, great care must be taken in design and application to limit or prevent viral escape, and target sequences for which escape has a fitness cost.
